# *cis*-(Acetato-κ^2^ *O*,*O*′)(5,5,7,12,12,14-hexa­methyl-1,4,8,11-tetra­aza­cyclo­tetra­decane-κ^4^ *N*,*N*′,*N*′′,*N*′′′)nickel(II) perchlorate monohydrate

**DOI:** 10.1107/S1600536812013232

**Published:** 2012-03-31

**Authors:** Tapashi G. Roy, Debashis Palit, Babul Chandra Nath, Seik Weng Ng, Edward R. T. Tiekink

**Affiliations:** a Department of Chemistry, University of Chittagong, Chittagong 4331, Bangladesh; b Department of Chemistry, University of Malaya, 50603 Kuala Lumpur, Malaysia; c Chemistry Department, Faculty of Science, King Abdulaziz University, PO Box 80203, Jeddah, Saudi Arabia

## Abstract

The complete cation in the title hydrated mol­ecular salt, [Ni(CH_3_CO_2_)(C_16_H_36_N_4_)]ClO_4_·H_2_O, is generated by the application of crystallographic twofold symmetry; the perchlorate anion and water mol­ecule are each disordered around a twofold axis. The Ni^II^ atom exists within a *cis*-N_4_O_2_ donor set based on a strongly distorted octa­hedron and defined by the four N atoms of the macrocyclic ligand and two O atoms of a symmetrically coordinating acetate ligand. In the crystal, hydrogen bonding (water–acetate/perchlorate O—H⋯O and amine–perchlorate N—H⋯O) leads to layers in the *ab* plane. The layers stack along the *c* axis, being connected by C—H⋯O(water) inter­actions. The crystal studied was found to be a non-merohedral twin; the minor component refined to 15.9 (6)%.

## Related literature

For background to macrocyclic complexes, see: Hazari *et al.* (2010 ▶). For a related structure, see: Roy *et al.* (2012 ▶). For the treatment of data from twinned crystals, see: Spek (2009 ▶).
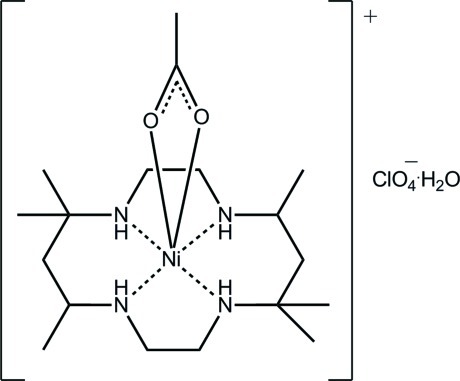


## Experimental

### 

#### Crystal data

[Ni(C_2_H_3_O_2_)(C_16_H_36_N_4_)]ClO_4_·H_2_O
*M* *_r_* = 519.71
Monoclinic, 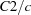

*a* = 9.4041 (2) Å
*b* = 15.9593 (4) Å
*c* = 16.0721 (6) Å
β = 96.534 (3)°
*V* = 2396.48 (12) Å^3^
*Z* = 4
Cu *K*α radiation
μ = 2.58 mm^−1^
*T* = 100 K
0.25 × 0.20 × 0.15 mm

#### Data collection

Agilent SuperNova Dual diffractometer with an Atlas detector
Absorption correction: multi-scan (*CrysAlis PRO*; Agilent, 2011 ▶) *T*_min_ = 0.850, *T*_max_ = 1.000
5502 measured reflections
2480 independent reflections
2286 reflections with *I* > 2σ(*I*)
*R*_int_ = 0.024

#### Refinement

*R*[*F* ^2^ > 2σ(*F* ^2^)] = 0.067
*wR*(*F* ^2^) = 0.162
*S* = 1.09
2480 reflections
171 parameters
45 restraints
H-atom parameters constrained
Δρ_max_ = 0.60 e Å^−3^
Δρ_min_ = −0.62 e Å^−3^

### 

Data collection: *CrysAlis PRO* (Agilent, 2011 ▶); cell refinement: *CrysAlis PRO*; data reduction: *CrysAlis PRO*; program(s) used to solve structure: *SHELXS97* (Sheldrick, 2008 ▶); program(s) used to refine structure: *SHELXL97* (Sheldrick, 2008 ▶); molecular graphics: *ORTEP-3* (Farrugia, 1997 ▶) and *DIAMOND* (Brandenburg, 2006 ▶); software used to prepare material for publication: *publCIF* (Westrip, 2010 ▶).

## Supplementary Material

Crystal structure: contains datablock(s) global, I. DOI: 10.1107/S1600536812013232/hb6703sup1.cif

Structure factors: contains datablock(s) I. DOI: 10.1107/S1600536812013232/hb6703Isup2.hkl

Additional supplementary materials:  crystallographic information; 3D view; checkCIF report

## Figures and Tables

**Table:** 

Ni—O1	2.118 (2)
Ni—N1	2.089 (3)
Ni—N2	2.136 (3)

**Table:** 

O1^i^—Ni—O1	62.28 (13)

Symmetry code: (i) 
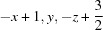
.

**Table 2:** Hydrogen-bond geometry (Å, °)

*D*—H⋯*A*	*D*—H	H⋯*A*	*D*⋯*A*	*D*—H⋯*A*
N1—H1⋯O2	0.88	2.47	3.289 (14)	154
N1—H1⋯O3^i^	0.88	2.35	3.181 (9)	158
N2—H2⋯O4^ii^	0.88	2.50	3.276 (8)	148
O1w—H1w1⋯O1^iii^	0.84	1.94	2.754 (11)	163
O1w—H1w2⋯O2	0.84	2.14	2.950 (18)	163
C3—H3*A*⋯O1*W*^iv^	0.99	2.14	3.057 (13)	153

Symmetry codes: (i) 
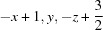
; (ii) 
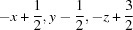
; (iii) 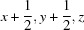; (iv) 
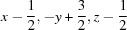
.

**Fig. 1.**
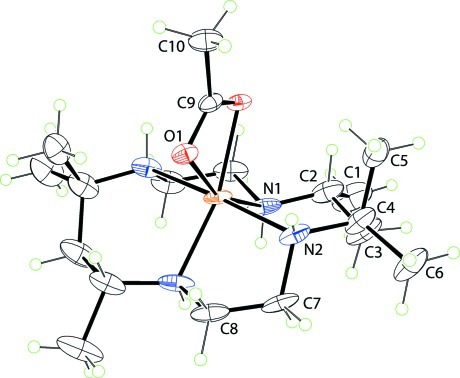
The molecular structure of the cation in (I) showing displacement ellipsoids at the 50% probability level. The cation has crystallographic twofold symmetry and unlabelled atoms are generated by the symmetry operation 1 - x, y, 3/2 - z.

**Fig. 2.**
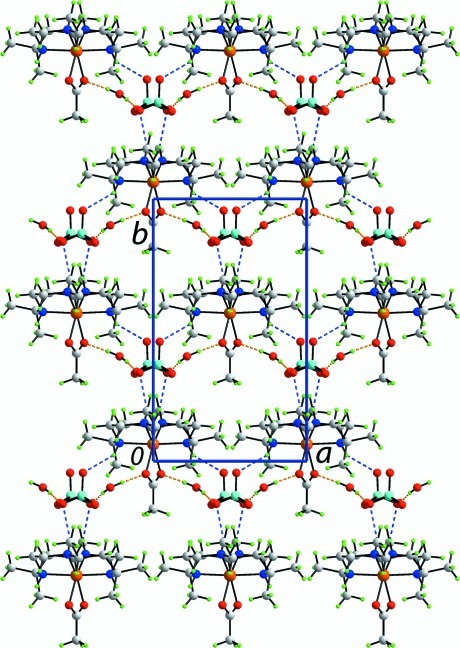
A view of the supramolecular layer in the ab plane in (I). The O—H···O and N—H···O hydrogen bonds are shown as orange and blue dashed lines, respectively. The illustrated perchlorate and water molecules are disordered about a twofold axis and each is present 50% of the time.

**Fig. 3.**
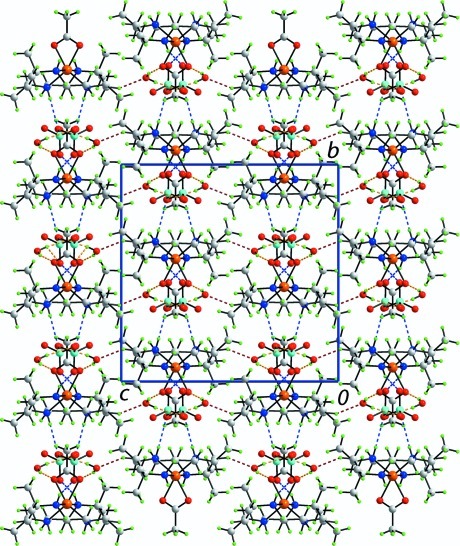
A view of the unit-cell contents in projection down the a axis in (I). The O—H···O, N—H···O and C—H···O interactions are shown as orange, blue and brown dashed lines, respectively. The illustrated perchlorate and water molecules are disordered about a twofold axis and each is present 50% of the time.

## supplementary crystallographic information

### Comment

As a continuation of systematic studies into the synthesis, characterization and biological activities of substituted tetraazamacrocyclic ligands and their metal complexes (Hazari *et al.*, 2010; Roy *et al.*, 2012), crystals of the title hydrated salt, (I), were isolated and characterized crystallographically.

The asymmetric unit of (I) comprises half a NiL(O_2_CMe) cation, Fig. 1, as this is has crystallographic twofold symmetry, half a perchlorate anion (this is disordered about a twofold axis) and half a water molecule of solvation (this is also disordered about a twofold axis); where *L* is 5,5,7,12,12,14-hexamethyl-1,4,8,11-tetraazacyclotetradecane. The Ni^II^ atom exists within a *cis*-N_4_O_2_ donor set defined by the four N atoms of the macrocyclic ligand and two acetate-O atoms, Table 1. The coordination geometry is based on an octahedron. There are significant distortions from the ideal geometry owing in part to the restricted bite angle of the acetate ligand as manifested in the O1—Ni—O1*^i^* angle of 62.28 (13)°; symmetry operation *i*: 1 - *x*, *y*, 3/2 - *z*. In particular, this bite angle restricts the putative *trans* O1—Ni—O1 angle to 158.84 (12)°; the N2—Ni—N2^i^ angle = 175.92 (17)°.

In the crystal packing, the water molecule forms O—H···O hydrogen bonds to the acetate-O1 and perchlorate-O2 atoms, while the amine-H atoms form hydrogen bonds to perchlorate-O atoms; the N1—H atom is bifurcated, Table 2. The hydrogen bonding leads to layers in the *ab* plane, Fig. 2. Layers are connected along the *c* axis by C—H···O(water) interactions, Fig. 3 and Table 2.

### Experimental

The title complex, (I), was prepared by the anion exchange reaction of [NiL(O_2_CMe)][O_2_CMe] with perchlorate, where *L* is 5,5,7,12,12,14-hexamethyl-1,4,8,11-tetraazacyclotetradecane. Thus, [NiL(O_2_CMe)][O_2_CMe] (0.495 g, 1.0 mmol) was dissolved in hot methanol (40 ml) and sodium perchlorate hexahydrate (0.460 g, 2.0 mmol) added. The reaction mixture was heated for 15 min. During heating a blue product separated out. After cooling at room temperature for 30 min, the product, (I), was filtered off, washed with methanol followed by diethyl ether and dried in a desiccator over silica-gel. Light-purple prisms of (I) were obtained from slow evaporation of its methanol solution. Yield 65%. *M*.pt: 512–513 K. Anal. Calc for C_18_H_41_ClN_4_NiO_7_: C, 41.68; H, 7.97; N, 10.81; Ni, 11.18%. Found: C, 44.53; H, 7.72; N, 10.78; Ni, 11.01%. FT—IR (KBr, cm^-1^): 1598 ν(O_2_C), 3202 ν(N—H), 2981 ν(C—H), 1369 ν(CH_3_), 1177 ν(C—C), 520 (Ni—N), 1126, 623 ν(ClO_4_).

### Refinement

The H-atoms were placed in calculated positions (O—H = 0.84, N—H = 0.88 and C—H = 0.98–1.00 Å) and were included in the refinement in the riding model approximation, with *U*_iso_(H) = 1.2–1.5*U*_equiv_(carrier atom). The perchlorate and water molecules are disordered across a twofold axis. The Cl—O bonds lengths were restrained to 0.01 Å of each other, as were the O···O contact distances. The anisotropic displacement parameters were restrained to be nearly isotropic. Finally, the methyl-H atoms of the acetate group are disordered over two positions of equal weight. The crystal studied is a non-merohedral twin. The twin domains were separated by the *TwinRotMat* routine in *PLATON* (Spek, 2009). The minor component refined to 15.9 (6)%.

### Crystal data

**Table:**

[Ni(C_2_H_3_O_2_)(C_16_H_36_N_4_]ClO_4_·H_2_O	*F*(000) = 1112
*M**_r_* = 519.71	*D*_x_ = 1.440 Mg m^−^^3^
Monoclinic, *C*2/*c*	Cu *K*α radiation, λ = 1.54184 Å
Hall symbol: -C 2yc	Cell parameters from 3218 reflections
*a* = 9.4041 (2) Å	θ = 5.5–76.4°
*b* = 15.9593 (4) Å	µ = 2.58 mm^−^^1^
*c* = 16.0721 (6) Å	*T* = 100 K
β = 96.534 (3)°	Prism, light-purple
*V* = 2396.48 (12) Å^3^	0.25 × 0.20 × 0.15 mm
*Z* = 4	

### Data collection

**Table:**

Agilent SuperNova Dual diffractometer with an Atlas detector	2480 independent reflections
Radiation source: SuperNova (Cu) X-ray Source	2286 reflections with *I* > 2σ(*I*)
Mirror monochromator	*R*_int_ = 0.024
Detector resolution: 10.4041 pixels mm^-1^	θ_max_ = 76.6°, θ_min_ = 5.5°
ω scan	*h* = −11→11
Absorption correction: multi-scan (*CrysAlis PRO*; Agilent, 2011)	*k* = −18→19
*T*_min_ = 0.850, *T*_max_ = 1.000	*l* = −3→20
5502 measured reflections	

### Refinement

**Table:**

Refinement on *F*^2^	Primary atom site location: structure-invariant direct methods
Least-squares matrix: full	Secondary atom site location: difference Fourier map
*R*[*F*^2^ > 2σ(*F*^2^)] = 0.067	Hydrogen site location: inferred from neighbouring sites
*wR*(*F*^2^) = 0.162	H-atom parameters constrained
*S* = 1.09	*w* = 1/[σ^2^(*F*_o_^2^) + (0.0529*P*)^2^ + 9.9029*P*] where *P* = (*F*_o_^2^ + 2*F*_c_^2^)/3
2480 reflections	(Δ/σ)_max_ < 0.001
171 parameters	Δρ_max_ = 0.60 e Å^−^^3^
45 restraints	Δρ_min_ = −0.62 e Å^−^^3^

### Special details

**Table:**

Geometry. All e.s.d.'s (except the e.s.d. in the dihedral angle between two l.s. planes) are estimated using the full covariance matrix. The cell e.s.d.'s are taken into account individually in the estimation of e.s.d.'s in distances, angles and torsion angles; correlations between e.s.d.'s in cell parameters are only used when they are defined by crystal symmetry. An approximate (isotropic) treatment of cell e.s.d.'s is used for estimating e.s.d.'s involving l.s. planes.
Refinement. Refinement of *F*^2^ against ALL reflections. The weighted *R*-factor *wR* and goodness of fit *S* are based on *F*^2^, conventional *R*-factors *R* are based on *F*, with *F* set to zero for negative *F*^2^. The threshold expression of *F*^2^ > σ(*F*^2^) is used only for calculating *R*-factors(gt) *etc*. and is not relevant to the choice of reflections for refinement. *R*-factors based on *F*^2^ are statistically about twice as large as those based on *F*, and *R*- factors based on ALL data will be even larger.

### Fractional atomic coordinates and isotropic or equivalent isotropic displacement parameters (Å^2^)

**Table:**

	*x*	*y*	*z*	*U*_iso_*/*U*_eq_	Occ. (<1)
Ni	0.5000	0.56446 (4)	0.7500	0.0300 (3)	
Cl1	0.4637 (3)	0.86782 (11)	0.71627 (15)	0.0559 (6)	0.50
O2	0.6083 (10)	0.8409 (9)	0.7174 (9)	0.197 (12)	0.50
O3	0.4105 (13)	0.8372 (5)	0.7910 (6)	0.111 (5)	0.50
O4	0.4585 (9)	0.9554 (3)	0.7181 (5)	0.085 (3)	0.50
O5	0.3800 (9)	0.8336 (6)	0.6466 (5)	0.111 (3)	0.50
O1W	0.7196 (13)	0.8995 (10)	0.8864 (7)	0.147 (6)	0.50
H1W1	0.7967	0.9187	0.8726	0.220*	0.50
H1W2	0.6729	0.8794	0.8433	0.220*	0.50
O1	0.4508 (2)	0.45087 (14)	0.80858 (16)	0.0318 (5)	
N1	0.5584 (4)	0.64503 (18)	0.6573 (2)	0.0459 (9)	
H1	0.5406	0.6967	0.6721	0.069*	
N2	0.2798 (3)	0.5692 (2)	0.6999 (2)	0.0412 (8)	
H2	0.2413	0.5227	0.7164	0.062*	
C1	0.5402 (7)	0.6902 (4)	0.5067 (4)	0.085 (2)	
H1A	0.6439	0.6833	0.5075	0.128*	
H1B	0.5188	0.7483	0.5206	0.128*	
H1C	0.4930	0.6766	0.4508	0.128*	
C2	0.4854 (5)	0.6312 (3)	0.5713 (3)	0.0546 (12)	
H2A	0.5042	0.5722	0.5546	0.066*	
C3	0.3231 (5)	0.6426 (3)	0.5688 (4)	0.0681 (16)	
H3A	0.2832	0.6489	0.5095	0.082*	
H3B	0.3059	0.6961	0.5973	0.082*	
C4	0.2374 (5)	0.5741 (3)	0.6076 (3)	0.0535 (12)	
C5	0.2620 (5)	0.4882 (3)	0.5708 (3)	0.0554 (11)	
H5A	0.3634	0.4733	0.5823	0.083*	
H5B	0.2345	0.4895	0.5102	0.083*	
H5C	0.2039	0.4465	0.5963	0.083*	
C6	0.0754 (5)	0.5945 (4)	0.5877 (4)	0.0749 (17)	
H6A	0.0558	0.6499	0.6102	0.112*	
H6B	0.0190	0.5521	0.6134	0.112*	
H6C	0.0493	0.5944	0.5269	0.112*	
C7	0.2205 (4)	0.6372 (3)	0.7477 (3)	0.0534 (12)	
H7A	0.1153	0.6310	0.7446	0.064*	
H7B	0.2415	0.6920	0.7229	0.064*	
C8	0.2843 (4)	0.6345 (3)	0.8372 (3)	0.0514 (11)	
H8A	0.2428	0.6799	0.8689	0.062*	
H8B	0.2614	0.5803	0.8624	0.062*	
C9	0.5000	0.4119 (3)	0.7500	0.0312 (10)	
C10	0.5000	0.3177 (3)	0.7500	0.0555 (16)	
H10A	0.4629	0.2973	0.8008	0.083*	0.50
H10B	0.5979	0.2973	0.7486	0.083*	0.50
H10C	0.4392	0.2973	0.7006	0.083*	0.50

### Atomic displacement parameters (Å^2^)

**Table:**

	*U*^11^	*U*^22^	*U*^33^	*U*^12^	*U*^13^	*U*^23^
Ni	0.0253 (4)	0.0131 (4)	0.0551 (6)	0.000	0.0195 (4)	0.000
Cl1	0.0651 (14)	0.0221 (8)	0.0840 (16)	0.0086 (8)	0.0232 (11)	−0.0024 (8)
O2	0.201 (15)	0.173 (14)	0.218 (15)	0.025 (9)	0.034 (10)	−0.029 (9)
O3	0.150 (9)	0.049 (5)	0.131 (8)	−0.031 (5)	0.013 (6)	0.048 (5)
O4	0.104 (7)	0.022 (3)	0.141 (8)	0.003 (3)	0.063 (5)	−0.004 (3)
O5	0.117 (7)	0.117 (7)	0.093 (6)	0.022 (6)	−0.005 (5)	−0.035 (5)
O1W	0.122 (9)	0.232 (15)	0.087 (7)	−0.090 (10)	0.017 (6)	−0.022 (8)
O1	0.0294 (12)	0.0186 (11)	0.0479 (14)	−0.0039 (9)	0.0068 (10)	0.0020 (9)
N1	0.0461 (19)	0.0204 (14)	0.079 (2)	0.0067 (13)	0.0404 (18)	0.0113 (14)
N2	0.0320 (15)	0.0318 (16)	0.063 (2)	0.0107 (13)	0.0194 (14)	0.0140 (14)
C1	0.090 (4)	0.070 (4)	0.106 (5)	0.023 (3)	0.058 (4)	0.052 (3)
C2	0.059 (3)	0.044 (2)	0.067 (3)	0.018 (2)	0.034 (2)	0.026 (2)
C3	0.061 (3)	0.065 (3)	0.083 (3)	0.034 (2)	0.029 (3)	0.044 (3)
C4	0.038 (2)	0.058 (3)	0.066 (3)	0.0198 (19)	0.0136 (19)	0.024 (2)
C5	0.041 (2)	0.070 (3)	0.054 (2)	0.014 (2)	0.0018 (19)	0.010 (2)
C6	0.044 (3)	0.094 (4)	0.087 (4)	0.032 (3)	0.010 (3)	0.031 (3)
C7	0.038 (2)	0.038 (2)	0.090 (3)	0.0211 (17)	0.033 (2)	0.014 (2)
C8	0.042 (2)	0.034 (2)	0.086 (3)	0.0081 (17)	0.042 (2)	0.002 (2)
C9	0.024 (2)	0.019 (2)	0.050 (3)	0.000	−0.001 (2)	0.000
C10	0.074 (4)	0.017 (2)	0.076 (4)	0.000	0.010 (3)	0.000

### Geometric parameters (Å, º)

**Table:**

Ni—O1	2.118 (2)	C2—H2A	1.0000
Ni—N1^i^	2.089 (3)	C3—C4	1.532 (7)
Ni—N1	2.089 (3)	C3—H3A	0.9900
Ni—O1^i^	2.118 (2)	C3—H3B	0.9900
Ni—N2	2.136 (3)	C4—C5	1.521 (7)
Ni—N2^i^	2.136 (3)	C4—C6	1.556 (6)
Cl1—O4	1.400 (5)	C5—H5A	0.9800
Cl1—O5	1.404 (6)	C5—H5B	0.9800
Cl1—O2	1.424 (8)	C5—H5C	0.9800
Cl1—O3	1.439 (7)	C6—H6A	0.9800
O1W—H1W1	0.8400	C6—H6B	0.9800
O1W—H1W2	0.8399	C6—H6C	0.9800
O1—C9	1.259 (3)	C7—C8	1.495 (7)
N1—C8^i^	1.481 (5)	C7—H7A	0.9900
N1—C2	1.488 (6)	C7—H7B	0.9900
N1—H1	0.8800	C8—N1^i^	1.481 (5)
N2—C7	1.475 (5)	C8—H8A	0.9900
N2—C4	1.493 (6)	C8—H8B	0.9900
N2—H2	0.8800	C9—O1^i^	1.259 (3)
C1—C2	1.534 (6)	C9—C10	1.503 (7)
C1—H1A	0.9800	C10—H10A	0.9800
C1—H1B	0.9800	C10—H10B	0.9800
C1—H1C	0.9800	C10—H10C	0.9800
C2—C3	1.532 (6)		
			
N1^i^—Ni—N1	104.01 (18)	C3—C2—H2A	108.2
N1^i^—Ni—O1^i^	158.84 (12)	C1—C2—H2A	108.2
N1—Ni—O1^i^	96.96 (11)	C2—C3—C4	118.2 (3)
N1^i^—Ni—O1	96.96 (11)	C2—C3—H3A	107.8
N1—Ni—O1	158.84 (12)	C4—C3—H3A	107.8
O1^i^—Ni—O1	62.28 (13)	C2—C3—H3B	107.8
N1^i^—Ni—N2	85.68 (14)	C4—C3—H3B	107.8
N1—Ni—N2	91.80 (13)	H3A—C3—H3B	107.1
O1^i^—Ni—N2	96.56 (11)	N2—C4—C5	107.7 (3)
O1—Ni—N2	86.94 (11)	N2—C4—C3	110.4 (4)
N1^i^—Ni—N2^i^	91.80 (13)	C5—C4—C3	112.0 (4)
N1—Ni—N2^i^	85.68 (14)	N2—C4—C6	111.1 (4)
O1^i^—Ni—N2^i^	86.94 (11)	C5—C4—C6	107.3 (5)
O1—Ni—N2^i^	96.56 (11)	C3—C4—C6	108.4 (4)
N2—Ni—N2^i^	175.92 (17)	C4—C5—H5A	109.5
O4—Cl1—O5	112.8 (5)	C4—C5—H5B	109.5
O4—Cl1—O2	109.7 (5)	H5A—C5—H5B	109.5
O5—Cl1—O2	109.9 (5)	C4—C5—H5C	109.5
O4—Cl1—O3	107.8 (4)	H5A—C5—H5C	109.5
O5—Cl1—O3	108.5 (5)	H5B—C5—H5C	109.5
O2—Cl1—O3	108.0 (5)	C4—C6—H6A	109.5
H1W1—O1W—H1W2	107.9	C4—C6—H6B	109.5
C9—O1—Ni	88.4 (2)	H6A—C6—H6B	109.5
C8^i^—N1—C2	113.0 (3)	C4—C6—H6C	109.5
C8^i^—N1—Ni	103.3 (3)	H6A—C6—H6C	109.5
C2—N1—Ni	116.1 (2)	H6B—C6—H6C	109.5
C8^i^—N1—H1	108.0	N2—C7—C8	110.3 (3)
C2—N1—H1	108.0	N2—C7—H7A	109.6
Ni—N1—H1	108.0	C8—C7—H7A	109.6
C7—N2—C4	113.9 (3)	N2—C7—H7B	109.6
C7—N2—Ni	103.7 (3)	C8—C7—H7B	109.6
C4—N2—Ni	120.9 (2)	H7A—C7—H7B	108.1
C7—N2—H2	105.7	N1^i^—C8—C7	110.0 (3)
C4—N2—H2	105.7	N1^i^—C8—H8A	109.7
Ni—N2—H2	105.7	C7—C8—H8A	109.7
C2—C1—H1A	109.5	N1^i^—C8—H8B	109.7
C2—C1—H1B	109.5	C7—C8—H8B	109.7
H1A—C1—H1B	109.5	H8A—C8—H8B	108.2
C2—C1—H1C	109.5	O1^i^—C9—O1	120.9 (4)
H1A—C1—H1C	109.5	O1^i^—C9—C10	119.6 (2)
H1B—C1—H1C	109.5	O1—C9—C10	119.6 (2)
N1—C2—C3	111.1 (4)	C9—C10—H10A	109.5
N1—C2—C1	112.4 (5)	C9—C10—H10B	109.5
C3—C2—C1	108.6 (4)	C9—C10—H10C	109.5
N1—C2—H2A	108.2		
			
N1^i^—Ni—O1—C9	−175.68 (14)	O1—Ni—N2—C4	−123.0 (3)
N1—Ni—O1—C9	11.9 (4)	C8^i^—N1—C2—C3	180.0 (3)
O1^i^—Ni—O1—C9	0.0	Ni—N1—C2—C3	60.9 (4)
N2—Ni—O1—C9	99.05 (14)	C8^i^—N1—C2—C1	−58.0 (4)
N2^i^—Ni—O1—C9	−83.05 (14)	Ni—N1—C2—C1	−177.1 (3)
N1^i^—Ni—N1—C8^i^	109.4 (3)	N1—C2—C3—C4	−73.5 (6)
O1^i^—Ni—N1—C8^i^	−67.8 (3)	C1—C2—C3—C4	162.3 (5)
O1—Ni—N1—C8^i^	−78.4 (5)	C7—N2—C4—C5	−161.4 (3)
N2—Ni—N1—C8^i^	−164.6 (3)	Ni—N2—C4—C5	74.0 (4)
N2^i^—Ni—N1—C8^i^	18.6 (3)	C7—N2—C4—C3	76.1 (4)
N1^i^—Ni—N1—C2	−126.3 (3)	Ni—N2—C4—C3	−48.5 (4)
O1^i^—Ni—N1—C2	56.5 (3)	C7—N2—C4—C6	−44.2 (5)
O1—Ni—N1—C2	45.9 (5)	Ni—N2—C4—C6	−168.8 (3)
N2—Ni—N1—C2	−40.3 (3)	C2—C3—C4—N2	65.0 (6)
N2^i^—Ni—N1—C2	142.9 (3)	C2—C3—C4—C5	−55.0 (7)
N1^i^—Ni—N2—C7	10.5 (2)	C2—C3—C4—C6	−173.1 (5)
N1—Ni—N2—C7	−93.4 (2)	C4—N2—C7—C8	−172.0 (3)
O1^i^—Ni—N2—C7	169.4 (2)	Ni—N2—C7—C8	−38.6 (4)
O1—Ni—N2—C7	107.7 (2)	N2—C7—C8—N1^i^	60.1 (4)
N1^i^—Ni—N2—C4	139.7 (3)	Ni—O1—C9—O1^i^	0.0
N1—Ni—N2—C4	35.8 (3)	Ni—O1—C9—C10	180.000 (1)
O1^i^—Ni—N2—C4	−61.4 (3)		

Symmetry code: (i) −*x*+1, *y*, −*z*+3/2.

### Hydrogen-bond geometry (Å, º)

**Table:**

*D*—H···*A*	*D*—H	H···*A*	*D*···*A*	*D*—H···*A*
N1—H1···O2	0.88	2.47	3.289 (14)	154
N1—H1···O3^i^	0.88	2.35	3.181 (9)	158
N2—H2···O4^ii^	0.88	2.50	3.276 (8)	148
O1w—H1w1···O1^iii^	0.84	1.94	2.754 (11)	163
O1w—H1w2···O2	0.84	2.14	2.950 (18)	163
C3—H3*A*···O1*W*^iv^	0.99	2.14	3.057 (13)	153

Symmetry codes: (i) −*x*+1, *y*, −*z*+3/2; (ii) −*x*+1/2, *y*−1/2, −*z*+3/2; (iii) *x*+1/2, *y*+1/2, *z*; (iv) *x*−1/2, −*y*+3/2, *z*−1/2.
